# Identifying veterinary surgeons’ barriers to, and potential solutions for, improving antimicrobial stewardship among sheep farmers in Northern Ireland

**DOI:** 10.1002/vro2.78

**Published:** 2024-04-12

**Authors:** Paul E. Crawford, Kim Hamer, Fiona Lovatt, Malgorzata C. Behnke, Philip A. Robinson

**Affiliations:** ^1^ Department of Animal Health Behaviour and Welfare Harper Adams University Newport UK; ^2^ School of Biodiversity One Health and Veterinary Medicine College of Medical Veterinary and Life Sciences Garscube Campus, University of Glasgow Glasgow UK; ^3^ School of Veterinary Medicine and Science Sutton Bonnington Campus University of Nottingham Nottingham UK; ^4^ Flock Health Ltd., Egglesburn Farm, Eggleston Barnard Castle UK; ^5^ Harper & Keele Veterinary School Harper Adams University Campus Edgmond UK; ^6^ Faculty of Natural Sciences Keele University Keele UK

## Abstract

**Background:**

In order to improve antimicrobial stewardship (AMS), including changes in antimicrobial prescribing and use, an enhanced understanding is needed of the barriers that veterinary surgeons (vets) encounter to institute such change.

**Methods:**

A qualitative approach, using grounded theory, was followed. Interviews and discussion groups, with vets and farm industry stakeholders in Northern Ireland (NI), were undertaken to identify and explore attitudes and behaviours surrounding AMS, with a particular emphasis on the barriers vets encountered and the context within which they were working.

**Results:**

Seven inter‐related themes associated with improving AMS among their sheep farming clients were identified. The first six addressed barriers were working under commercial and practical constraints, farmer behaviour, multiple medicine sources, poor prescribing practice, a perceived lack of incentive or facilitation to improve AMS and a perceived lack of action by regulators to challenge poor AMS. The seventh theme revealed suggestions vets considered that may improve AMS in NI, including greater state intervention in recording and regulating medicine sales.

**Conclusions:**

Improving AMS will require vets and their client farmers to change behaviour. This will involve concerted effort over an extended period of time to enact and embed change. Veterinary surgeons believe that further action by the industry and state to develop centralised antimicrobial sales recording and by the state to enforce prescribing regulations will aid their efforts. However, critical to achieving this is the development of a sustainable and funded mechanism to create more meaningful farmer–vet consultation around flock health prior to every prescription to improve AMS and sheep welfare.

## INTRODUCTION

All antibiotics authorised for use in farmed livestock in the UK are prescription only medicines (POM‐V).^1^ This provides veterinary surgeons (vets) with a pivotal and unique role in managing the supply of medicines and a focal point for statutory control.^1^, ^2^ Antibiotic use in livestock, it has been proposed, increases selection pressure, driving bacterial antimicrobial resistance (AMR), with potential spill‐over affecting human healthcare^3^, ^4^, ^5^, ^6^ causing increased morbidity and mortality.^6^, ^7^ Additionally, AMR development could affect the success of treatment of livestock diseases, increasing morbidity and mortality.^8^ Thus, regardless of the weight of evidence linking animal use to AMR in the human population, vets have a vested interest in preserving antibiotic efficacy. These concerns have resulted in a wide range of regional and sectoral action plans to improve antimicrobial stewardship (AMS) in human and animal medicine.^9^, ^10^, ^11^

Statutory control of veterinary antibiotic prescribing in the UK falls under the Veterinary Medicines Regulations (VMR) and is achieved through inspectors appointed by the Secretary of State, or in the case of Northern Ireland (NI), appointed by the Department of Agriculture, Environment and Rural Affairs or the Department of Health (DoH), or acting jointly.^1^

The competitive nature of veterinary practice, whereby a client who does not obtain the medicines they demanded and simply seek out an alternative veterinary practice who will meet their demands has been highlighted.^3^, ^5^, ^12^, ^13^ The lack of formal consultation, before prescribing antibiotics to bona fide farming clients, has been reported previously.^5^

Benchmarking veterinary medicine use to identify intervention points, set targets and measure progress requires farm‐specific information and is central to AMS.^3^, ^6^, ^11^ European studies have reported that central, statutory recording of medicine prescribing, or sales data, with advisors having access to that information, combined with rigorous state enforcement of prescribing regulations, can significantly improve AMS.^13^, ^14^ Prescribers in the UK lag behind much of Europe, in access to such data or regulatory enforcement, with data collection on antimicrobial use in the sheep sector particularly poor,^15^, ^16^ with nothing specific to the NI sheep sector.

The objective of this study was to use qualitative methods to gain enhanced understanding of the vet's role in influencing NI flock owners to improve AMS, and the barriers they encounter, with particular emphasis on their role as prescribers.

## MATERIALS AND METHODS

The data presented here are from a wider mixed‐methods project considering medicine use in the NI sheep flock. Following an explanation of the programme and assurances of confidentially, consent was sought for electronic recording of interviews and discussion groups. Contemporaneous written notes were made where consent to electronically record was not obtained, or recording was not possible. All interviews and discussion groups were undertaken by the first author, a sheep breeder and vet with 20 years of professional experience, who was known to some of the participants.

Individual interviews were undertaken with 15 vets (Table 1) and 13 industry stakeholders (ISs) between December 2021 and July 2022. Interviews were predominantly conducted via internet video calls^17^ due to Covid 19‐related restrictions,^18^ although, when permitted, participants were offered the choice of face‐to‐face or internet video call interview. Veterinary surgeons, all of whom worked in mixed‐large animal general practice in NI, were recruited using convenience sampling and by ‘snowball’ sampling through recommendations from other interviewees,^19^ with no exclusion criteria applied. Industry stakeholders were all recruited by approaching them directly, because of their known or perceived relevance to the overall project aims.

**TABLE 1:** Summary of the veterinary surgeons who participated in interviews.

Code	Sex	Age (under or over 50 years)	Location (county)	Interview length (min)	Interview transcript length (words)
V01	Female	Under (U)	A	50	8300
V02	Male	U	A	69	4200
V03	Male	O (>50 years)	B	34	5400
V04	Male	U	C	51	3900
V05	Female	U	C	50	6300
V06	Female	U	D	58	8500
V07	Male	O	E	79	6700
V08	Male	O	F	84	15,100
V09	Female	U	F	41	5900
V10	Male	O	E	70	9200
V11	Male	U	C	31	4200
V12	Male	O	C	44	5400
V13	Male	U	C	57	2500
V14	Male	O	E	43	6500
V15	Male	U	D	58	8100

*Note*: No incentive or remuneration was provided to any interview participant. Location was represented by a letter assigned to each of the six counties of Northern Ireland in order to protect participant anonymity.

A semi‐structured approach to one‐to‐one interviewing was adopted. An interview guide was prepared for vets (Appendix S1), based on themes emerging from semi‐structured interviews undertaken with 27 NI sheep farmers by the first author, as part of wider project. Semi‐structured interviews with ISs were individualised based on their area of expertise and influence and undertaken between January and November 2022.

Five discussion groups were convened between September 2022 and February 2023 for vets and ISs (Table 2). A convenience sample of those willing to participate was used throughout recruitment, with further ‘snowball’ sampling.^19^ A semi‐structured guide for the discussion groups was based on the themes emerging from the earlier interviews (Appendix S2).

**TABLE 2:** Summary of discussion groups.

	Participants	Format	Recording	Duration (min)
DG1 (October 2022)	Five veterinary surgeons (one previously participated in an interview)	Face‐to‐face with a meal provided	Recorded electronically and transcribed in full	89
DG2 (January 2023)	Seven veterinary surgeons (four previously participated in an interview)	Online video conference	Recorded electronically and transcribed in full	80
DGP (October 2022)	Six representatives of six companies in the pharmaceutical trade actively engaged with large animal veterinary practice (none participated in an interview)	Face‐to‐face	Recorded electronically and transcribed in full	46
DGR (January 2023)	Two representatives of the red meat promotion sector (one previously participated in an interview)	Online video conference	Contemporaneous notes	
DGL (February 2023)	Six representatives of farming lobbying and representative organisations (none participated in an interview)	Online video conference	Contemporaneous notes	

*Note*: The only participants that received any incentive were the participants of DG1 who received a meal during their discussion group session.

All recorded interviews and discussions were transcribed in full and, along with contemporaneous notes from the remaining interviews and discussions, coded and analysed utilising Nvivo 10 software.^20^ Themes were identified from the data in an iterative, rather than the data being sorted against a pre‐defined list of themes (see Appendix S3 for more details of the grounded theory approach).^21^

## RESULTS

All the vet interviews (V) were recorded (mean 55 min, range 31–84 min). Interviews with seven of the 13 ISs were recorded (mean 48 min, range 16–61 min). Contemporaneous notes were taken during the remaining interviews. Five discussion groups were held, two for vets (DG1 and DG2) and three for other ISs (DGP, DGR and DGL) (Table 2).

The viewpoint of the vets in relation to prescribing and their influence on AMS on NI sheep farms is reported here, with specific insight from other ISs which frames the context in which the vets worked. Additional exemplar quotes identified from interviews and discussion groups, to corroborate views and experiences presented below in narrative form, are available in Appendix S4.

**FIGURE 1:**
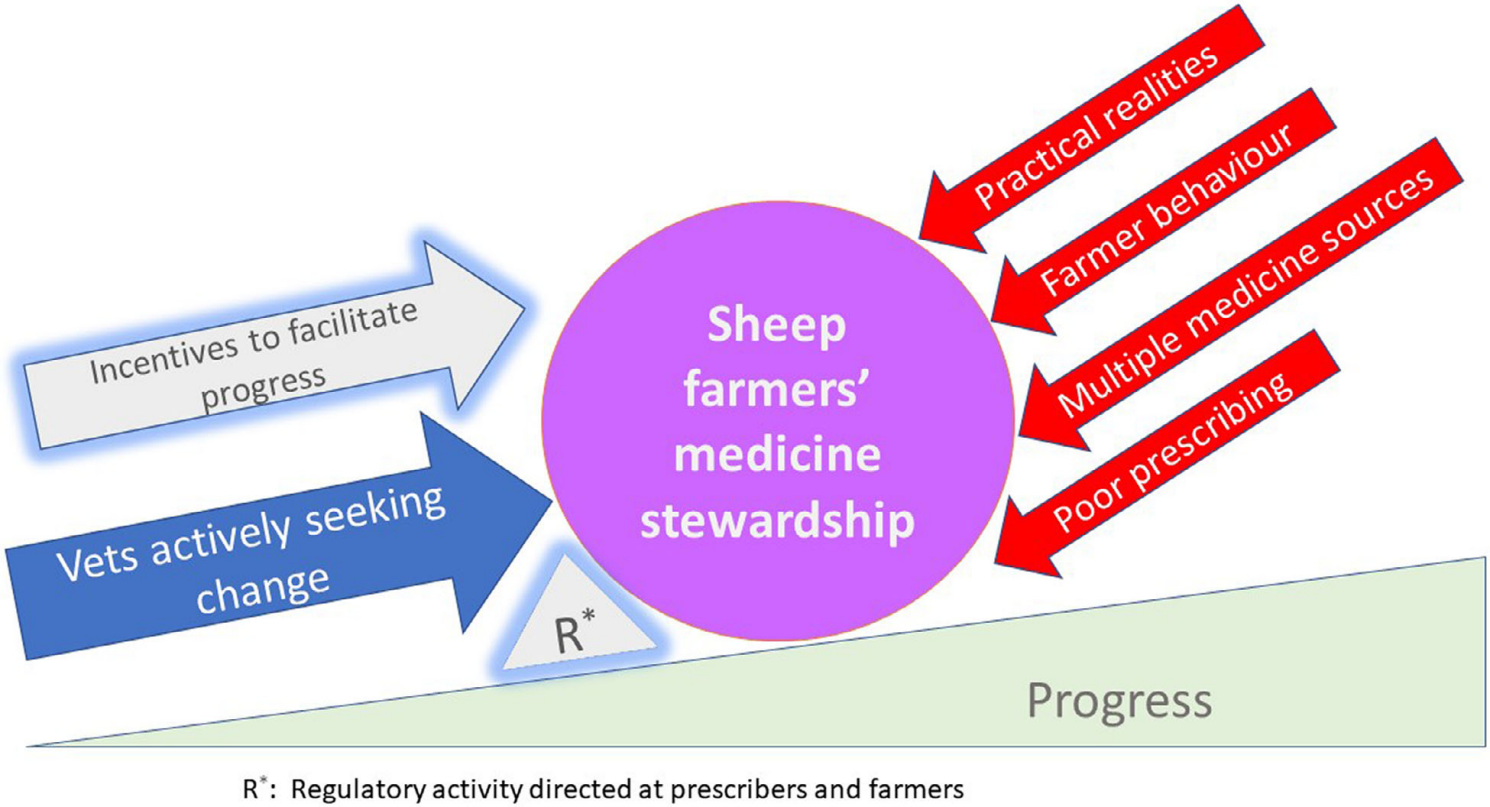
Forces influencing antimicrobial stewardship (AMS) in Northern Ireland in relation to management of sheep flocks. Forces acting against Northern Irish (NI) vets in their ongoing, uphill struggle to improve AMS among NI sheep farmers working under practical realities, farmer behaviour, multiple medicine sources, poor prescribing practice and a perceived lack of action by regulators to challenge poor AMS. They also perceived the lack of incentives or facilitation, that they felt should be in place, to aid them and farmers to improve AMS.

Analysis of the comments from vets during interviews and subsequent discussion groups, conducted through an iterative process of reading, coding and reflection,^22^ identified seven inter‐related themes associated with improving AMS among their sheep farming clients. Six themes concerned barriers to AMS were working under commercial and practical constraints, farmer behaviour, multiple medicine sources, poor prescribing practice, lack of incentive or facilitation and lack of action by regulators (Figure 1). The seventh theme concerned ‘Vet's solutions’. Further details about the themes are provided below.

1. **Working under commercial and practical constraints**
  Veterinary surgeons identified that commercial and practical realities affected their businesses and interactions with farming clients. These included the need for their business to make a profit, including from medicine sales, poor on‐farm facilities and the low‐profit margins their farming clients achieved. They cited examples of how advice was given for free. This included low‐cost improvements in husbandry and hygiene, which could form an important intervention to improve AMS.
  V10: Anyone who hasn't had a vet on farm within the past sort of 6–12 months, we are doing visits, free of charge.
  DG1: It comes back to commercial choice. Selling the stuff (medicines) makes money, not selling the stuff doesn't make money.
  DG1: We are doing very few anthelmintic sales. To be totally honest, anyone who comes seriously to talk about it, I tell them what to get and where to get it because all we can do is advise. We can't compete on price.
  Finally, vets and others identified that, disregarding any production losses, simply purchasing medicines to treat preventable conditions was a significant financial burden for some farms.
2. **Farmer behaviour**
  Veterinary surgeons identified a range of farmer behaviours, which impacted on their prescribing practice. Central to these was farmers’ unwillingness to seek or accept professional, preventative advice a priori, instead responding only in the face of a disaster.
  DG1: Farmers definitely need to have an abortion disaster to get them interested in using vaccine and their memories are short—that is, they often abandon vaccination later, despite the disaster.
  Veterinary surgeons recognised that they were not the only information source that farmers used to garner advice on the care of their flock and not all such advices were constructive. However, where suitable farmers could be identified to promote good practice, vets recognised the positivity of peer‐to‐peer mentoring.
  DG2: We had a farmer who bought some pedigree sheep from somebody who works for the state's agricultural department. They told him the only thing to keep lambs right was a shot of enrofloxacin at birth. I refused to sell it to him, but he was going to get it somewhere because this woman told him it was what to do.
  Decision making surrounding anthelmintic use was an area, which caused participating vets particular frustration. Veterinary surgeons described how there was limited but increasing interest from farmers for faecal egg count tests. Farmers, vets reported, also had strong habitual preferences for certain anthelmintics, including routine treatment of young lambs with adult flukicides.
  DG2: The standard first dose for lambs is Levafas diamond (3.0% [w/v] levamisole hydrochloride and 6.0% [w/v] oxyclozanide, Norbrook Pharmaceuticals). A fluke product in a lamb a month or 6 weeks old! There is an obsession with Levafas diamond!
  Veterinary surgeons recognised that they only had limited control over medicine use once it left their practice, with farmers failing to follow prescribing direction and diverting medicines prescribed for cattle into sheep.
  DG1: Your guess is as good as mine whether they give a course or a single dose of Pen Strep (procaine penicillin [200 mg/mL] and dihydrostreptomycin sulphate [250 mg/mL], Norbrook Pharmaceuticals). I would say they just take notions.
  Veterinary surgeons also described pressure from farmers; this could take the form of the farmer demanding certain medicines, or becoming threatening when their medicine requests were turned down. Veterinary surgeons also outlined other pressure that arose from the availability of medicines from other sources.
  DG1: I was surprised to find when spectinomycin (50 mg/mL) oral solution (Ceva Animal Health) went off the market, that, … I have never seen hissy fits like it with some of those farmers. You had some pedigree boys [sic] literally lost the absolute plot. F'ing and blinding and stamping up and down saying they would get it some way. It was just wild the behaviour of some of them. (Editor note: oral antibiotic such as spectinomycin was widely used prophylactically in neonatal lambs—a practice that is no longer recommended over and above drug access.)
3. **Multiple medicine sources**
  Veterinary surgeons unanimously described farmers sourcing medicines from multiple sources; legal and occasionally, illicit. Veterinary surgeons reported some farmers were open in disclosing this to them, on other occasions vets spotted bottles or packaging on‐farm during visits.
  DG1: I have observed that there is a bit of a black market, possibly also counterfeit medicine. The farmers where I work now aren't as daft as to tell us, but the practice I used to be in, they would tell you no bother! A lot of the bottles I've seen, the printing and colour isn't quite right. Blurry.
  DG1: We're getting people telling us ‘Ah, we jagged it with that (tilmicosin 300 mg/mL injection) and it hasn't worked’. And we realise that we haven't supplied them with any.
4. **Poor prescribing practice**
  Veterinary surgeons participating in this study reported behaviours in themselves or other vets they knew, that negatively impacted AMS. Individually, vets outlined strategies they were employing to counter some of the negative behaviours.
  V06: If the farmer knows what he wants it is normally just given to him without any questions asked.
  DG1: You cannot get the people using injections of long‐acting oxytetracycline to prevent abortion to stop. *Facilitator asks: Why do you prescribe in this situation?* I don't think I am allowed not to [by my employer]. (Editor note: abortion can be associated with a number of infectious causes including *Chlamydophila abortus*, where tetracycline antibiotics may be used—hence, routine prophylaxis is not recommended.)
  DG2: Having explained to our clients how to manage their flocks without resorting to an alternative prophylactic antibiotic, following Spectam's (spectinomycin [50 mg/mL] oral solution, Ceva Animal Health) withdrawal from the market, you hear there is a practice not too far up the road that is apparently making their own version of Spectam. So, we are wasting our time in some respects.
  Basic information about their clients’ flocks, such as flock size, was unknown to many of the vets.
  DG1: I'd say there is a fair significant percentage of my sheep clients where I've never seen their sheep up close.
  IS06: Vets getting accurate information on the actual numbers on the sheep side is more difficult than with cattle because you can't pull quantity information from APHIS (APHIS is a Northern Ireland Food Animal Information System) such as cattle. You are requiring a count being made by the farmer themselves. So, you are into self‐declaration, which has its limitations.
5. **Lack of incentives or facilitation**
  Veterinary surgeons identified the need to incentivise and facilitate progress on AMS, including knowledge transfer. They identified making time and receiving adequate remuneration as facilitators for delivering more farmer training or advisory visits. The lack of incentives available in NI and the benefits these have brought elsewhere were recognised by IS (see Appendix S4).
  DG2: There are various grants out there for things. Why not give a grant for weighing scales? There should be an electric weigh scale on every sheep farm.
  V07: It was actually astounding what we discovered about our clients and what they discovered about us when we had 2 or 3 h to spend with them and somebody else was paying.
6. **Lack of action by regulators**
  Veterinary surgeons indicated they had a strained working relationship with state authorities and they believed a comparable situation existed between some farmers and the state. Regardless, they called for further regulation of prescribing either through state mechanisms or quality assurance schemes. Veterinary surgeons highlighted a lack of understanding of the current regulatory process and unfamiliarity about where to turn if they identified a concern. Veterinary surgeons identified tighter regulation of medicines in the Republic of Ireland.
  DG1: Anyone who suggests there is a partnership between private vets and the state authorities is deluded.
  DG1: The idea of writing a health plan down scares farmers. That is something the Department (state authorities) might see. Documenting things on their farm that are other than perfect. I would say all farmers have a proactive fear of the state's agriculture department; how they can give (farming support) and take away (fines and penalties).
  V07: Somebody can have a bulk milk tank (antibiotic) failure or have a medicine raid by the state's agriculture department and there is no joined up thinking ask from where or whom did the farmer got the medicines that caused the residue failure.
  Two pertinent statements were made by ISs, reiterating the viewpoint of vets that lack of enforcement of the VMR was currently an area of concern.
  IS05: The problem with the enforcement model is that it is not resourced and it is all over the place and the different branches of the state need to stop fighting among ourselves. The Veterinary Medicines Directorate had delegated enforcement to the DoH and the DoH wants to walk away from this. So, at the moment there is no one enforcing anything in NI and no one making legislation.
  IS06: Our biggest issue is the state's agriculture department don't have the resource to, or power to ever get a drug residue, identified in meat, through to conviction.
7. **Veterinary surgeons’ solutions**
  Veterinary surgeons were forthcoming with individual success stories and suggestions on changes that they considered may help achieve improved AMS in NI; however, they also indicated that such progress was going to require a continual and concerted effort by all sectors to initiate and embed change. Veterinary surgeons were keen to explore the introduction of centralised medicine recording for their farm clients. Any such scheme, vets considered, would need to be backed by regulatory activity to ensure records of every medicine purchase would be available to the vet providing flock health advice or completing an antibiotic audit. They reported that a voluntary medicine recording and analysis scheme currently active in NI had potential to deliver better insight into on‐farm medicine use; however, significant problems with its management at state level had already led to several of the participants abandoning it. Individually vets recounted situations where they, or collectively their practice, had achieved progress in promoting AMS.
  V19: We tried for a long time to sell calf pneumonia vaccines and invested an awful lot of time with very little return and then eventually found the right farm, who agreed to take it on. He really needed to take it on, they had a serious problem. And it spread like wildfire from that, you know, just by word of mouth.
  DG2: I think some form of centralised prescription recording is going to have to be done, or something similar, to focus minds on the fact that these are prescription medicines. So farmers and vets treat them as such.
  Veterinary surgeons, however, were divided on the merits of farmers being forced to only obtain veterinary services from one vet.

## DISCUSSION

Veterinary surgeons working with sheep farmers in NI described a constant up‐hill battle, as they navigated a complex series of inter‐related barriers to achieving better AMS among their sheep farming clients. These could be visualised as forces opposing the attempts of vets to improve AMS (Figure 1). Farmers reverting to previous behaviour were recognised; thus, constant re‐iteration of the messages on appropriate medicine use and preventative behaviours was required. This is time‐consuming, although vets identify it as achievable when they are able to have direct and specific conversations with engaged farmers who are open to learning. Identifying it was not only farmers who needed to modify some behaviours, vets recognised that the process of changing their behaviour took time and focused effort,^13^ creating discomfort, until they gain confidence in their new working practices.

Our results confirm previous findings, where vets are unable to meet every request for medicine with a detailed consultation,^5^ either through a lack of veterinary resources or through farmers’ unwillingness to engage. The VMR can only be followed in full with positive interaction from both parties and without undue coercion being applied.^13^ Veterinary surgeons reported that they felt pressurised to prescribe antibiotics, including from their colleagues, where they did not consider the farmer's request clinically efficacious or in keeping with good AMS principles.^22^, ^23^ Complacency has previously been recognised in prescribing behaviours,^23^, ^24^ this may account for some of the poor prescribing that vets identified among themselves and their peers. They also felt frustration that any efforts they made to improve AMS were negated by the actions of other vets and the ability of farmers to ‘shop around’. Together, these experiences reflected previous reports in veterinary and human medical prescribing,^8^, ^23^, ^25^ adding further detail to the debate about how to progress interventions aimed at improving AMS.

Veterinary surgeons reported farmers were reluctant to reduce habitual or precautionary medicine use in case it adversely affected production. They were also concerned that restrictive prescribing could adversely affect their vet–client relationship, as reported in other veterinary sectors.^5^, ^8^, ^12^ Some vets also reported they, or their colleagues, had a similar fear of adverse consequences, if they were to adopt more restrictive prescribing.^23^ When combined with the availability of medicines from multiple sources, this creates a situation in which the diligent prescriber feels frustrated and conflicted as they know that farmers can source medicines elsewhere that they may not want to prescribe. Previous calls for regulation to square this circle, addressing the issues created by commercial competition,^5^, ^13^ was strongly echoed by vets in this study. This call for greater regulation comes despite an apparent breakdown in relations between private practice vets and state authorities. The alleged failure of the state to provide effective, proportionate and visible regulation may affect further improvement in AMS.

Previous publications have highlighted difficulties in maintaining accurate on‐farm records^26^ and the benefits of centralised antimicrobial prescription recording to monitor both end‐user behaviour and that of prescribers.^14^ Accurate medicine use data have been recognised as being central to the effective AMS tools of target setting and benchmarking.^27^ These previous findings, alongside the multiple sources of medicines vets recognise on farms and poor farmer record keeping, suggest a centralised database of prescriptions or medicine sales records, automatically uplifted at time of prescription, or sale, is required in NI. Combined with accurate data on flock size and structure, which vets identify as a gap in their prescribing knowledge, could facilitate vets’ understanding of on‐farm medicine use. They could then confidently guide farmers to improve AMS through eliminating inappropriate or unnecessary medicine use and develop preventative healthcare strategies, understanding the totality of medicine use on the farm. Centralised recording may also offer opportunities for automated feedback to vets on their prescribing behaviour and to benchmark this nationally.^13^, ^28^

Despite this need to make profit, vets indicated that they were giving their professional advice away, often for free,^29^ feeling unable to charge for advisory services and seeing medicine purchases, based on their advice, being transacted by other businesses. Farming businesses were making low‐profit margins on sheep farming in NI at the time of this study,^30^ leaving limited funds for the up‐front costs of preventative care,^8^, ^12^, ^13^ or critical infrastructure, such as weigh crates to administer medicines accurately. Ultimately, finances play an important role in the complex pressures on vets and their farming clients and act to limit veterinary manpower and, thus, time available to deliver consultations and farm advisory visits. As private businesses, farm animal practices need to make a profit on medicines sales and professional fees for services and advice,^31^, ^32^ drivers which have previously been shown to influence prescribing.^23^ High rates of failure to sustain change have been recognised in human medical care^33^; however, repetition of AMS messages, particularly one‐to‐one, with reflection, has been shown to increase long‐term adoption of change.^34^, ^35^ Someone, therefore, must remunerate the profession for their services—the farmer, medicine sales profit or funding from the public purse.^13^ This could facilitate vets to engage and re‐engage with their sheep farming clients, on an ongoing and recurrent manner, to drive forward AMS, particularly given its importance in limiting the development of AMR.^6^, ^31^, ^32^ In addition, a multi‐faceted approach to modifying veterinary medicine use and prescribing,^5^, ^13^, ^25^ will be required to address the heterogenicity of both vets’ beliefs and behaviours and farmers’ responses to the veterinary engagement described here and previously recognised.^29^, ^37^, ^38^ This needs to, in particular, challenge habitual behaviour based on preference rather than best practice.^23^

Finally, it is important to highlight that vets were able to recount incidences when clients were open to, and adopted, their mutually agreed preventative strategies to minimise need for therapeutic medicines. This reflects others’ reports of the farmer–vet relationship and its potential to improve AMS.^8^, ^13^, ^34^

Limitations to this study include the potential for participant bias because vets with a particular interest or point of view on prescribing practice may have volunteered to take part, or indeed may have declined to participate, knowing that their practices were sub‐standard or even illegal. Nonetheless, a heterogenicity of views were expressed, for example, on the question of one‐farm‐one‐vet, where diametrically opposed views were presented. Further studies, including quantitative elements, may be needed to fully understand the penetration of differing viewpoints before firm policy ideas can be proposed.

It is clear that vets want to be confident they have full disclosure of all medicines used on farm, although it is a question for the agri‐food industry to consider how centralised medicine recording could be developed, funded and the underpinning data‐sharing defined. Higher standards and more rigorous implementation of existing regulations have been called for, to ensure all prescribers work to the same standard. State authorities were called to work proactively together, and in constructive relationship with vets and farmers, to investigate poor prescribing and illicit sources of medicine.

Veterinary surgeons need to take additional steps to ensure that they have sufficient information, to prescribe responsibly, and that their clinical reasoning is recorded. To facilitate this, farmers will have to accept a new approach, whereby vets seek to consult more closely before prescribing medicines and engage in ongoing planning to ensure flock health. These changes will require concerted effort over a period of years to establish new habits in both sectors.

We believe, based on the data presented here, that increasing income from delivery of regular advisory services could be critical to ensuring the future viability of the veterinary sector in NI and to continually drive improvements in AMS. How this increased income is derived is a matter for urgent debate among the wider agri‐food industry and the state.

## AUTHOR CONTRIBUTIONS

All authors contributed to the design of the wider project from which these data are drawn. Paul E. Crawford conducted all data collection and analysis. All authors contributed to the writing and critical review of the manuscript.

## CONFLICTS OF INTEREST STATEMENT

The authors declare they have no conflicts of interest.

## ETHICS STATEMENT

The authors confirmed that the ethical policies of the journal, as noted on the journal's author guidelines page, have been adhered to. Ethical approval was obtained from the Research Ethics Committee of Harper Adams University, under approval number 0010‐202101‐PGMPHD.

## Supporting information

Supporting Information

## Data Availability

Due to the confidentially agreements entered between researcher and participants, the raw data cannot me made available.
