# Supplementary material for: Identifying veterinary surgeons’ barriers to, and potential solutions for, improving antimicrobial stewardship among sheep farmers in Northern Ireland
Source: Vet Rec Open. 2024 Apr 12;11(1):e278. doi: 10.1002/vro2.78 (PMC11014869; doi:10.1002/vro2.78)
Supplement: Supplementary file 1 — Supporting Information [file VRO2-11-e278-s001.pdf]

Supporting Information

Appendix S1

Interview guide used for fifteen veterinary surgeon interviews.

Protocol for sales	• Are agreed flock health plans on file for staff to dispense against<br>• Generalised dispensing guide for lay staff<br>• Questioning reason for medicine request<br>• Follow-up diagnostic / preventative<br>• Requests presented over the counter to a person other than a qualified prescriber<br>• Farm stock level knowledge
Different approaches for specific antibacterials & indications / uses	• Critical antibiotics<br>• Oral products for neonatal lambs;<br>• Antibiotic injection for abortion,<br>• Soluble power for footbath
Understanding & interpretation of Royal College of Veterinary Surgeons / Veterinary Medicines Directorate etc. guidelines	• Under care<br>• Competence for safe use<br>• Questions that should be asked
Role of / place for national treatment guidelines	• Sustainable control of parasites of sheep - SCOPS<br>• Lameness 5-point-plan<br>• British Small Animal Veterinary Association equivalent schemes
Personal and practice/business level involvement in national training schemes	• MilkSure (https://www.bcva.org.uk/content/milksure)<br>• Farm Vet Champions (https://knowledge.rcvs.org.uk/amr/farm-vet-champions/?&&type=rfst&set=true#cookie-widget)<br>• Your knowledge?
Beliefs about farmers knowledge when requesting drugs	
Services offered, uptake	On farm
Belief and frustrations about (in)ability to influence farmer behaviour	Carrots and sticks
National initiatives; future direction	• Existing<br>• Feared<br>• Hoped for
Spectam (spectinomycin) – did you see an effect?	

<u>Notes and comments</u>

One follow-up question was sent by email to participating veterinary surgeons with a brief introduction to explain the request and a reassurance that the confidentiality of the participants would be respected.

When interviewing and participating in discussion groups, little mention was made by vets of corticosteroid use in sheep but when I reviewed the farmer supplied medicine records they appear in half of these record.

Why are these drugs prescribed, or are they requested by farmers?

As ever, anything you say will be confidential and anonymised before reporting in my thesis and other outputs. Also, happy to have a chat on the 'phone / zoom if you prefer.

Appendix S2 - Discussion group (DG) guides

Vets' discussion group guide (DG1 and DG2)

Thank participants for help in interviews, recruiting farmers and supplying records	
Highlights of research to date	• Medicine use – patterns, low v high, prescribing patterns, treat rather than prevent, lameness ongoing issues, pockets of progress, metrics<br>• Flock health plans or flock health planning
Questions / discussion points	• Resources – vets, impact of part-time farming, to deliver flock health planning and prescribing, testing, and prescribing of anthelmintics<br>• Resources – IT to manage prescribing<br>• One-farm-one-vet? or Central prescription register or what?<br>• Unlicensed drugs – recurrent sales
Successes	transition to vaccine over antibiotic for abortion, pain-relief

Lobbying organisation representatives' discussion group guide (DGL)

Thank participants for agreeing to host and facilitate a Zoom for my PhD with some of your colleagues.	
I present research findings to date around	• medicine use<br>• lack of oversight in medicine (antibiotic) supply<br>• flock health planning<br>• Farm Quality Assurance Scheme – opinions of and behaviour relating to inspections and record keeping<br>• medicine recording on-farm use<br>• impact of (non-disclosed of) sourcing medicines from multi-vet practice<br>• perceived lack of incentives in sector to progress<br>• ongoing lameness problem, low awareness of control plans or even causes<br>• gaps in research base<br>• and from a positive standpoint reduction in antibiotic for abortion already achieved and the world carried on when Spectam (spectinomycin) disappeared - showing the industry can respond positively to animal health messages
Seek feedback on what industry might think are appropriate ways to progress any of these areas (these following options are not my recommendations at this point but are options that have been raised by others and or are in use elsewhere)	• 1 farmer 1 vet<br>• improved medicine recording [currently a statutory requirement for all, not just a FQAS standard, but not enforced to any degree]<br>• improving oversight of prescription of medication (antibiotic)<br>• prescription register [which any certifying vet would have access to a record of all medicine purchased]<br>• test results required to prescribe anthelmintics<br>• future farm support tied to participation in schemes; Southern ewe premium, English Pathway, Scottish… not got a name yet but one suggestion is payment linked to data-recording, discussion, and knowledge exchange events.<br>• Where do Business Development Groups (or their successor) sit in all this?<br>• Incentivising and resourcing farmers – doing this without ignoring the ones who have already taken steps to improve<br>• diagnostic testing facilities<br>• Improved screening of livestock entering marts
Any other bits you think I should be asking questions about but have not done so to date	

Discussion groups for all other industry representatives (DGP (pharmaceutical industry) and DGR [red meat trade])

As above, plus, for those involved in quality assurance scheme management
• use of shower systems<br>• what goes in the medicine book is not all that goes in the sheep<br>• lack of incentive means [small] issues with inspections / inspectors means farmers throw the head up<br>• lack of progress on lameness

Appendix S3 – Details of the approach used to code interviews and discussion group outputs

This study forms part of a larger project considering medicine use in the Northern Ireland (NI) sheep flock.

The overall project employed a mixed-methods research approach. Elements of both quantitative and qualitative inquiry were used, providing both quantitative data, for example on quantities and types of medicine used, as well as qualitative data collection concerning the more subjective attitudes and reported behaviours of both farmers and vets as they described their experience of medicine use and stewardship in the NI flock.

The approach to the collection and analysis of the data followed a Grounded Theory approach, initially described by the American sociologists Glaser and Strauss in the 1960s as a methodology to develop theory from data.[1] There have been various adaptations to their original theoretical approach by researchers in the following decades, each of which may stress or draw from sometimes differing philosophical assumptions.[2] Some of these developments have introduced contradictions and outright disputes between authors,[3] and, as such, there is neither a single clear-cut definition of Grounded Theory, nor precise prescription for the assumptions to be followed with this methodological approach. Classic grounded theorists suggest that, at first, researchers should literally ignore the literature pertaining to the area under study,[3] difficult for a researcher from a natural science background to achieve due to prior scientific knowledge and engagement with the subject area. Nonetheless, the emphasis was to look into the collected data with little or no preconceived ideas, letting the data tell its own story, rather than the researcher testing pre-conceived hypotheses. When collecting and analysing the data in this study of medicines use, it was therefore important to remain openminded about the findings and implications of the data collected in the project.

A scoping survey,[4] targeted at NI sheep farmers, was the initial data collection tool. This provided initial insights into the knowledge and behaviours of NI sheep farmers and informed the development of an interview guide for the interviews with sheep farmers. Semi-structured interviews with 27 NI sheep farmers were undertaken between July 2021 and March 2022, alongside the quantitative analysis of medicine records obtained from 52 NI sheep farmers.[5] The outline of the approach to the analysis of the vet interviews in this paper also reflects the process undertaken during the conduct and analysis of the farmer interviews.

All interviews and discussion groups were undertaken by the first author, who then transcribed all electronic audio recordings. This transcription was undertaken as soon as practical after the interview, and, with the exception of days when more than one interview was scheduled on the same day, before subsequent interviews. The transcripts were then transferred to the Nvivo software package to facilitate coding. Coding was also undertaken in parallel to interviewing and transcription. When developing codes, the transcripts were re-read multiple times and initial codes, on occasion, were split into sub-sections or closely related codes combined to maintain a representative but manageable code set. Thus, each individual dataset was analysed iteratively and inductively from within the data and not against a predefined coding list, in a manner not dissimilar to reflective thematic analysis.[6]

Theoretical sampling is the concept that researchers base subsequent sampling on theories emerging from the data collected to date. This sampling is undertaken to both understand the breadth of the viewpoints as well as to explore them in greater detail.[3] Thus, initial interviews with farmers directed both the questions and type of farmer sought for subsequent interviews, as well as the wider participant groups to be studied. Other stakeholders engaged to participate following the insights gained from farmer interviews included the vets reported here alongside industry stakeholders such as those involved in red meat promotion, state officials and quality assurance schemes to gain greater insight into the themes emerging from the farmers.

It was difficult to achieve the optimal levels of theoretical sampling, at times, in the current project. This related to the obvious inability to force farmers, vets or other stakeholders to participate. In particular, it was difficult to deeply probe some suggestions put forward by vet participants about lax prescribing practices and illicit medicine sources. In effect, to interview vets engaged in either activity would require them to self-identify as such to the researchers and willingly talk on the record, albeit with a promise of anonymity, about such behaviours. To mitigate this, where possible, triangulation was used, to confirm reports of poor prescribing or illicit behaviour, from a range of stakeholders over several interviews and discussions.

This iterative approach of concurrent data collection, initial analysis and finding new participants based on the findings continued throughout the period of interviews, with coding notes taken for two purposes. Firstly, the notes informed the adjustment of subsequent interviews, where less focus was centred on areas where no new ideas were emerging and more focus was placed on newly emerging themes. Secondly, these notes,

or in classic grounded theory terminology, memos, served as the initial basis for identifying the themes emerging from, and between, the individual datasets.

Following the interviews with vets, central themes of the overall project were considered and the farmer interviews revisited and coding refined and a series of sector specific and cross-cutting themes recognised and described; this fed into the discussion groups and ultimately to the final project step, a multi-sectoral, in-person forum.

Finally, the end product of the Grounded Theory, a theory to describe, explain and predict behaviours surrounding medicine use in the NI flock, will only be possible to describe when all datasets are analysed, including the analysis of the quantitative elements of the data corpus, and the emerging themes woven together. As such, the findings of the paper presented here are not the final output of our approach considering medicine use in the NI sheep flock, but represent a significant part of that process, and will be combined with nested case-studies, and the viewpoints of NI sheep farmers, in an effort to develop theory to guide future policymaking on medicine use in sheep.

References

1 Glaser BG, Strauss AL. The Discovery of Grounded Theory: Strategies for Qualitative Research. Aldine Transactions, New Brunswick, NJ, USA; 1967.

2 Denzin NK. Grounded and Indigenous Theories and the Politics of Pragmatism. Sociological Inquiry 2010;80:296-312. https://doi.org/10.1111/j.1475-682X.2010.00332.x

3 Timonen V, Foley G, Conlon C. Challenges When Using Grounded Theory: A Pragmatic Introduction to Doing GT Research. Int J Qual Methods. 2018; 17:1609406918758086. https://doi.org/10.1177/1609406918758086.

4 Crawford PE, Hamer K, Lovatt F, Robinson PA. Sheep scab in Northern Ireland: Its distribution, costs and farmer knowledge about prevention and control. Prev Vet Med. 2022;205:105682. doi: 10.1016/j.prevetmed.2022.105682.

5 Crawford PE, Hamer K, Lovatt F, Behnke MC, Robinson PA. Improving analgesia provision for sheep: An analysis of farm medicine records and attitudes towards pain relief on sheep farms in Northern Ireland. Vet Rec Open. 2023;10:e75. doi: 10.1002/vro2.75.

6 Braun V, Clarke V. Using thematic analysis in psychology. Qual Res Psychol. 2006;3:77-101. DOI:10.1191/1478088706qp063oa.

Appendix S4 - Additional exemplar quotes

Anonymisation codes
DG1, DG2 – discussion groups for vets
DGP – discussion group for members of the pharmaceutical industry
DGR – discussion group with members of the red-meat processing and promotion sector
DGL – discussion group with members of the agricultural lobbying and industry representation sector, with a suffixed numeral to indicate different participants were an extract of a conversation between two or more participants if included
V – veterinary surgeon interviewee
IS – industry stakeholder interviewee

1 Working under commercial and practical constraints

DG2 Farmers don't even have scales to weigh sheep. So, I don't know how they know they are giving the correct dose of anything to any of their animals.

DG1 Poor lambing hygiene would feed into the number of ewes getting antibiotics. A lot of that would be reduced if we were doing things clean in the first place.

V06 I suppose the way I have got round it, the way used to get round it now, I put it into money terms. I tell them if you are spending 400 pounds on wormer, if you cut that out it will be 200, you are only going to spend half the money on wormers compared to what you would spend regularly.

V13 The farmer who has a lot of CODD (contagious ovine digital dermatitis) and is having to use a lot of macrolides to treat that, you would prefer if he didn't have CODD and he didn't have to use the macrolides and they are expensive, especially the long-acting ones, so he would prefer, if I can get a control measure for CODD without having to use macrolides then I am open to having a conversation about that.

DG1 I think they have no concept as to how much they actually spend on these medicines. I mean, when they have a really bad outbreak, if they actually realised how much money they had spent on drugs they would have a different view on the value of prevention.

P10 He had come to get drugs, I don't know what he came to get and then we went for a free visit, and I was walking around and I said 'Your sheep, half of them are bald and they are scratching against the fence'. And he was, yea, I had noticed they had been doing that. This was his first summer with sheep.

DGL Antibiotics are being used to prevent a risk to health and profit. Aging infrastructure on farms does not help with being in a position to thoroughly disinfect between batches etc.

DGL To help focus farmer's minds on the benefits of a preventative approach, helping farmer to appreciate the monetary costs of disease might help.

DGP I think in my limited experience it comes down to cold hard cash and as you said it is easier to write them off livestock than consider vaccine plans. If you look at, there is more money in milk so the farmers there are perceived to have more money to invest in that sort of thing and I think there is more of a willingness, they are in their vets regularly. I know when I was working in practice, sheep farmers you didn't see them unless they had a sheep that they couldn't get lambed or something like that. It was the only time that you saw them. They weren't people that they had vets out regularly on farm work.

2 Farmer behaviour

V06 If the lamb is in the crush and hasn't been dosed, it needs to be dosed. Again, quite a lot of them won't listen to you, because when the ewes are in the pen, they seem to need to worm them too!

V13 Some people just want a quick fix and a bottle to fix it rather than a management change to fix it. And that is difficult.

V15 I had a pedigree guy who started telling me he wasn't going to use Spectam [spectinomycin 50 mg/ml oral solution, Ceva Animal Health] any longer, someone who he met somewhere had told him to shoot Marbocyl [marbofloxacin 20 mg/ml, Vetoquinol] down their throat. A ml of 2%.

DG2 There are a lot of sheep farmers that see cattle and sheep as ruminants, and you use the same medicine in each.

DG2 The withdrawal of Spectam [spectinomycin 50 mg/ml oral solution, Ceva Animal Health] at the minute is causing an issue, with people on the 'phone looking for Spectam. 'I don't have it, doesn't exist'. *Farmer replies: "You are lying to me; you are keeping it for somebody else."* 'No, I reiterate, it doesn't exist'.

DG1 If you don't give then the antibiotic they request, someone else down the road will. It is difficult. You

just try to limit it and try not to give out too much higher end [Category B and C] antibiotics where you can.

DGL By the end of lambing, you get tired and lax, and that is when problems start with regard to things like scour. You started using oral antibiotics and then the next year you think I'll just use this from day one and it becomes a habit.

DGP We are all slow to change. We are all creatures of habit. So, look at that behavioural change. What motivates that behavioural change. You see with oral antibiotics for lambs. The behaviour changed when they were withdrawn. Guys were pulling their hair out 'oh my business is going to stop'. But we quickly adapted so, there is different motivators there. If we can work out the motivator, how do you get that guy to reflect and do something different.

DGP I think you touched on something there. When you said that farmers told to you that they felt that vets weren't interested in sheep. I think there is a lot of farmers not interested in vets. In the practice sheep farmers don't bother with engaging their vets for health plans.

DGP Farmer's view of lameness is a good example. If you are a non-farmer, you could maybe see a lame sheep quicker than the farmer. There is a level of lameness now which is almost acceptable on some farms. So, it is getting across those barriers. Not a simple task.

3 Multiple medicine sources

V07 It has certainly taken enough time for me to change my behaviours and I think vets have to be seen to be making the change and too many of us are unwilling or unable to do it.

V12 Farmers have pushed back on calls to use only one vet. Oh no, we want to use two or three different vets. And the LMC [Livestock Marketing Commission, who also oversee the main farm quality assurance scheme for sheep farmers in NI] have said that's OK, you can use two or three different vets. We are going to need records from all three. You will have to disclose it. I think that is the starting point.

V13 I often said this would be a good way to reduce the use of Spectam (spectinomycin) or even if you want to reduce the use of CIAs [critically important antibiotics] would be to stop supplying them. Because if they are not there, they cannot use them. You get this difficulty, and I am sure you have had this conversation with other vets, antibiotics in Northern Ireland are pretty readily available from a number of sources, not just your own nominated vet.

V13 We don't sell critical antibiotics for cows with *E. coli*, but the practice down the road sells it. And they just hand it out even though said farmers aren't clients.

V07 I have seen that situation when I am testing and some of the sheep are being lambed in the same shed you are testing in, bottles of things from various places including over the border, so that, those guys, I suppose, never get anything off of us.

DGR Obtaining services and medicines from multiple vets is OK under current standards but this has to be (self) disclosed and evidence provided that each vet has engaged with the farm in the past year.

4 Poor prescribing practice

DG1 To minimise inappropriate use of antibiotics that are required to be given daily, a lot of the farmers at our practice are prescribed long-acting amoxicillin products.

V02 We try to have conversations when we are selling antibiotics but again there are times it is busy and you end up giving them a bottle and you don't have the discussion and don't know why they needed it.

V06 It is a wee bit more iffy if a farmer asks for Marbocyl (marbofloxacin) or he asks for Cobactin (cefquinome) or he asks for Advocin (danofloxacin) there is a wee bit more resistance there. Again, it does depend on the farmer who asks as well. There are some big farmers or farmers who have been there a long time, they are asking for it normally, no questions asked, it is handed out to them. If a farmer came in a just asked for pen strep or he asks for Terramycin or he asks for Hexasol (flunixin meglumine with oxytetracycline) yea, probably 90% of the time it would be handed over to him.

V10 You know, you are only getting through to a few farmers, and there are lots of sheep farmers that we never come across because they just get on with it themselves.

V13 we have some sheep farmers that we never see if a farmer came for a bottle of Pen and Strep, the front counter staff will say, yes, what are you treating with it, what is it for and it will be dispensed to them.

DGP You know how products are sold to sheep farmers by vets. And it is sold, not prescribed. And that is why farmers, they develop habits. Whatever they have used, whatever dose rate it may have been a point in time where it was of some use and they haven't changed.

Opposing views were expressed over the approach taken in some other countries and proposed by some for Northern Ireland.

DG1 I think the idea of one vet per farm is a bad idea. It is anti-competitive. Whether we like it or not competition among us is what makes us better.

DG2 I think [tighter prescribing rules outlines already in force in other EU countries] that is something that should be welcomed.

V12 in an ideal world a farmer would have to be registered to a practice.

DGL One farm one vet should be resisted on many levels – medicine purchase choice, range of skills vets in different practices possess, competition.

DGP Any country which has applied the national veterinary prescribing system as it would be applied eventually in the Republic of Ireland, it changed behaviour overnight. And that changed the habits of the prescribing vet to you know, prescribe the right dose and so on and so forth. It is a game changer.

5 Lack of incentives or facilitation

DG1 You would need a big financial incentive possibly to make it formal flock health planning and medicine reviews happen.

DG2 Do we ever get state money for flock health planning? It has always been promised that is the problem, but we probably would all like to do that job.

IS13 *Discussing a recent long-term programme in Wales to engage farmers in health planning:* The money the scheme provided covered the vet visits and each year the farmer also had a pot of money specifically for lab fees so they can test. That was a massive incentive for them because we have seen a lot of farmers now testing and they say they wouldn't have considered testing for say, metabolic profiling before. We have loads of farmers doing that now where they wouldn't have done it before. Money is a massive incentive.

IS05 *Discussing the incentives available to encourage sheep farmers to participate in quality assurance schemes and associated medicines audits:* Is there a financial benefit right in their face? No. Not at this stage. Is there a stick to convince them they must do it at this stage? Not a very sharp one.

DGR It was recognised that there is currently no reward for many sheep farmers to participate in quality assurance schemes in NI. Intangible benefits are present but hard to use as a selling point. It was also recognised that some moves to improve medicine stewardship may not provide an economic return for the farmer and production benefits, if any, may not be seen for a period of time – again a hard sell to farmers. There is a need to resource the sheep sector with farmer-vet interaction time, funded in some way. This has to be part of a long-range plan so funding doesn’t just dry up again.

DGL Grant support historically has been pitched towards items or too high a value to be of interest to the majority of flock owners who typically have up to a couple of hundred sheep and work off farm.

DGP So, the problem with the recent state sponsored flock health planning initiative was there was a big drive for health plan and then serology or some sort of blood testing and then there was nothing.

DGP There are not carrots but there needs to be a carrot for this improved AMS (antimicrobial stewardship) and sheep health to work. Because, essentially you need a retailer saying actually, we can't have, we just can't have welfare issues. You are the processor. You need to come up with a strategy here that the lambs that are arriving at your facility there has been evidence that they have been, for example, vaccinated for footrot, if it is deemed necessary. Full stop.

DGP I would imagine, if it were me and I was coming back to the drawing board, it would be right back, retailer level, processor level. Put the push onto farmers but with the carrot not the stick and working back up the chain that way. Because, otherwise, and I am not being disrespectful to the vets in the room, but if you are relying on the vets to have the conversation with the farmers or the farmers to have the conversation with the vets, you are not going to get very far. You need something to drive that or facilitate that. Some sort of a premium scheme where you are paid extra for the lambs. Either paid extra or deducted if they are not up to the specification. Which every way. One is a carrot; one is a stick. I'd go carrot. It is the only thing that drives habit or changes habits. The problem with premium scheme is that you have to convince the consumer that paying the premium is a good idea.

6 Lack of action by regulators

DG2 The quality assurance inspector in the Republic of Ireland will check first that each product listed in the medicine record is licensed for sheep and if it is not the farmer is sent back to the prescribing vet as to why it was used. It has probably focused not only the farmers but I would say the vets' attitude.

V07 The Republic of Ireland, it seems that things have changed a lot - from a proper wild west situation to a fairly strictly regulated situation. They are properly afraid of the Department doing a raid and they do keep the records they need to keep.

V12 I would like to see the whole medicines thing better regulated. Probably even through the quality assurance end of things. The state's agriculture department has a role to play there too.

V12 We have a state agriculture department that is totally unfit for purpose in my opinion at this stage.

7 Vets seeking solutions

DG2 The whole voluntary centralised electronic medicine recoding and analysis tool, as rolled out in NI is a bit of a shambles. In theory I have had the system for two years and it and I've got no use out of it because getting permissions through DAERA [Department of Agriculture, Environment and Rural Affairs] to get the animal data (age, sex etc.) downloaded for the cattle side of things is a shambles. So, I just forgot about it because it was so poorly backed up. I know the LMC are offering money to try and engage vets, and are trying to push it hard and the whole thing has fallen pretty flat. The big issue is DAERA. It is getting access to APHIS [the state's livestock information service that records all cattle births, deaths, and movements]. You get forms to fill in with all these numbers than nobody seems to know what they are. It is not, there is herd number, business number and then some other number you send in and then DAERA loose the paperwork and you are no further forward. That is what I did I sent a pile of them in, DAERA have no record of them, and I just ran around in circles. The system prompters kept sending me bills for software which I didn't pay because I had nothing out of it. I got a free tablet out of it. That was the only thing.

V13 We certainly have fewer farmers now using prophylactic antibiotics for control of enzootic abortion. I think farmers that are vaccinating and now understand that the vaccination is the superior way to prevent it. I would give the credit for that to my late colleague. He was a sheep farmer and he believed passionately in the use of vaccine and I remember him at veterinary meetings standing up and saying that and he was speaking very much from his personal experience and he said it with conviction because he was saying it as a veterinary surgeon with an interest in sheep as well as a sheep farmer. We found that people would have respected his view on things because he had sheep of his own and he was a noted pedigree sheep breeder. He would have been a very strong advocate for getting away from long-acting tetracycline and to vaccinate instead and he would have made farmers very aware that was the way they should be doing it. I think that that message did get across. He instilled this belief into the younger vets in the practice, through conversations with he had with them.

V13 I spent maybe an hour on the farm and we talked about snatching calves, we talked about colostrum, we talked about hygiene and we talked all these things and we talked measuring, you know, you think just because your calf has got enough colostrum doesn't mean the total protein is right, you have to measure it or we don't know. I sort of felt I had got somewhere with it, you know, I thought yea, they did this, and they got their own refractometer and I helped make a bit of difference there. But you don't always get successes like that but it is nice when somebody feel that what you said is right and they have taken on board what you have said.

V15 While few farmers properly engage with the annual medicine review as part of their quality assurance, we did have people who we had the conversation with who were using too much, too many critically important antibiotics. And they genuinely have taken it on board, others are probably still using the same amount but not recording it.

V19 I think we do fairly well on educating in general with our clients. We talk about lameness. There is a big farm merchant two miles down the road. They sell Footvax cheaper than we can buy it. You know, so we do up the handouts for the dos and don'ts with Footvax and hand them out and give them all the advice, and the odd person buys Footvax from us. But, by in large, we do all the groundwork and keep them right on the Footvax but we don't make an awful lot of money out of it. We do very well on the enzootic abortion vaccines. We do very well with that in the autumn. We would have a good penetration I would say there now.

We achieved this success in abortion vaccine sales following years of hammering the message at the farmers. One thing I have learnt is you won't sell something to a farmer here. You won't do it directly, you know. They have to hear it off their neighbour.

For sheep abortion we have run meetings and on top of that we have prepared a three-page flyer, just the dos and don'ts and keeping it realistic, the expectations of vaccination and where the pitfalls and hurdles and how much to expect in your first year and so on. And once we got through that there, last autumn was quite phenomenal. We were running out of storage space. You have to order the vaccine in advance of the farmer demand, and people in this part of the world usually buy their replacements in at the last minute. Then they are in a big panic for the vaccines. I would nearly say, apart from that one, I would nearly say now, that we have everybody vaccinating now that we would like to vaccinate or that we would expect to

vaccinate, so, I'd be happy enough there. That 3-page flyer that we did, the information leaflet, was critical. People came in with all their questions, and you can speak to them over the counter but it is too much information for one conversation. You can say, that this home, read it and come back with your questions. Another big difference here is enzootic abortion vaccine became very affordable over the last few years. Hasn't happened with toxoplasmosis vaccine, uptake of which is still much lower.

V20 Things like abortion vaccines, I would say, 80% plus. Even the hill boys are vaccinating. We really pushed that a few years ago. We went from very little sales to massive sales and now a very high percentage of our clients would now be vaccinating for Enzo and Toxo. I think people realised you could spend six quid to vaccinate for both and save a lot of money and have more lambs to sell at the end of the day. Even people, anyone would have vaccinated if they had an abortion storm, but now even people who have not had an abortion storm, have talked to friends. Farmers listen to each other. I find that.

DGL DGL2 outlined how a local vet has started to work proactively with the interested sheep farmers in his area. They have developed a flock health group which has regular meetings and through it the participants can access discounts, deals on vaccine purchase etc. They also highlighted the work of the NSA (National Sheep Association) to provide accessible quality educational material on the web for farmers, young or old to access when it suits them best. DGL3 added that the annual health review with the vet is time and money well spent, even if they don't find anything wrong when blood tests are done it is time and money well spent knowing you are going in the right direction.

DGP I think that everybody tried to get the education awareness out there but ultimately there are still better ways and you need to find solutions and educate farmers better or get that message to farmers better. I see that with the lameness vaccine all the time. People start using Footvax and they didn't know it existed up to now. So, is that our fault that we are not getting our message out or is it the vet's fault or the practice that we are not getting that message out. I am not sure.
